# Essential amino acid supplements ingestion has a positive effect on executive function after moderate-intensity aerobic exercise

**DOI:** 10.1038/s41598-023-49781-z

**Published:** 2023-12-19

**Authors:** Kento Dora, Hayato Tsukamoto, Tadashi Suga, Keigo Tomoo, Asuka Suzuki, Yusuke Adachi, Masamichi Takeshita, Yumiko Kato, Mika Kawasaki, Wataru Sato, Akira Imaizumi, Sachise Karakawa, Hirohisa Uchida, Takeshi Hashimoto

**Affiliations:** 1 Faculty of Sport and Health Science, Ritsumeikan University, 1-1-1 Nojihigashi, Kusatsu, Shiga 525-8577 Japan; 2 Department of Biomedical Engineering, Toyo University, Kawagoe, Saitama Japan; 3 Faculty of Sport Sciences, Waseda University, Tokorozawa, Saitama Japan; 4 Department of Nutrition Science, Purdue University, West Lafayette, IN USA; 5 Institute of Food Sciences and Technologies, Ajinomoto Co., Inc., Kawasaki, Kanagawa Japan; 6 Research Institute for Bioscience Products & Fine Chemicals, Ajinomoto Co., Inc., Kawasaki, Kanagawa Japan; 7 Sports Nutrition Department, Ajinomoto Co., Inc., Chuo-ku, Tokyo, Japan

**Keywords:** Lifestyle modification, Nutritional supplements

## Abstract

Aerobic exercise acutely improves cognitive function (e.g., executive function (EF); memory recognition (MR)) and increases circulating brain-derived neurotrophic factor (BDNF). In addition, branched-chain amino acids (BCAA) ingestion acutely shortens the choice reaction time and increases brain BDNF. We examined whether the ingestion of essential amino acid (EAA) supplements (mainly composed of BCAA) would positively impact on cognitive function and circulating BDNF after moderate-intensity aerobic exercise. Twenty-two healthy young men received either an EAA supplements or the placebo (PL) 30 min before undergoing aerobic exercise. The participants performed a cycling exercise at 60% of peak oxygen uptake for 30 min. EF after aerobic exercise was better after the EAA treatment than after the PL treatment (*P* = 0.02). MR (*P* = 0.38 for response accuracy; *P* = 0.15 for reaction time) and circulating BDNF (*P* = 0.59) were not altered by EAA supplements. EF improvement was correlated with increases in some amino acids (leucine, isoleucine, valine, lysine, phenylalanine; all *P*s < 0.05) that are potential substrates for synthesizing neurotransmitters in the brain. These results suggest that EAA supplements ingestion had a positive effect on EF after moderate-intensity aerobic exercise, while MR and BDNF were not altered.

## Introduction

Preventing cognitive decline is essential to maintain quality of life^1,2^. It has been well established that habitual exercise is a useful strategy to improve cognitive function^3,4^. The benefits of habitual exercise on cognitive function are attributed to the repeated, acute improvements in cognitive function in response to exercise^5,6^. Importantly, the degree to which cognitive function is improved by acute exercise is associated with the cognitive improvement induced by chronic exercise training^6^. Therefore, exploring ways to increase the extent of acute improvements in cognitive function may be useful for developing an effective regular program to improve cognitive function and hence brain health. Nutritional supplementation may also be a feasible and useful method to enhance aerobic exercise-induced improvements in cognitive function, but little information on its effects is available^7^. Thus, exploring the additive effects of supplemental nutrients on exercise-induced cognitive improvements could suggest various strategies for maintaining brain health.

In recent years, the favorable effects of ingesting essential amino acid (EAA) supplements on the brain have been demonstrated^8–12^. Long-term ingestion of EAA supplements drastically reversed the inflammatory response and brain atrophy in a tauopathy model^9^ and counteracted neurotransmitter deficiency^10^. In addition, in a clinical trial targeting adults 55 years of age or older, 12 weeks of EAA supplements ingestion improved attention and cognitive flexibility^11^. Valine, leucine, and isoleucine, which are components of EAA supplements, are classified as branched-chain amino acids (BCAA). The ingestion of BCAA supplements acutely shortens the choice reaction time before aerobic exercise (30 min after ingestion) and during aerobic exercise^12^, but it is unclear whether BCAA supplements acutely enhance exercise-induced improvements in higher-order cognitive function (e.g., executive function [EF] and memory). In addition, cognitive decline may also be associated with systemic brain-derived neurotrophic factor (BDNF) levels^13^. BDNF is mainly synthesized in brain tissues and promotes neurogenesis, synaptic plasticity, and cell survival, particularly in the cerebral cortex and hippocampus^14^. A single bout of aerobic exercise increases brain BDNF levels and BDNF release into the blood^15^. Indeed, repeated acute increases in systemic BDNF levels (e.g., chronic exercise training) can improve cognitive function^16^, suggesting that the acute increase in BDNF levels may be a mechanism underlying habitual aerobic exercise-enhanced cognitive function^16,17^. Interestingly, acute administration of BCAA transiently increases BDNF levels in the rat brain^18^, and long-term BCAA injections increase hippocampal BDNF levels in mice^8^. However, it remains unclear whether aerobic exercise-induced increases in systemic BDNF levels are further enhanced by the ingestion of EAA supplements mainly composed of BCAA.

In this study, we examined the additive effect of EAA supplements on improvements in cognitive function induced by moderate-intensity aerobic exercise as well as increases in systemic BDNF levels. We hypothesized that EAA supplements ingestion would have a positive effect on exercise-induced improvements in higher-order cognitive function and increase circulating serum BDNF levels.

## Results

### Changes in serum BDNF levels

There was a significant main effect of time on the serum BDNF level (*P* < 0.01), indicating that the serum BDNF level was higher at pre-EX (*P* < 0.01) and post-EX (*P* < 0.01) than at baseline (Table 1). However, no significant main effect of treatment (*P* = 0.44) or significant treatment × time interaction (*P* = 0.59) were observed.

**Table 1:** Heart rate, perceived exertion, and blood data throughout EAA and PL treatments.

	Time points								*P* values		
	Baseline	Pre-EX	5 min	10 min	15 min	20 min	25 min	30 min (i.e., post-EX)	Treatment	Time	Interaction
HR (bpm)			ab	abc	abcd	abcde	abcde	abcdefg			
EAA	60.6 ± 8.3	60.0 ± 7.9	130.7 ± 11.6	140.0 ± 12.1	146.7 ± 12.9	150.6 ± 12.4	152.1 ± 13.1	155.6 ± 14.6	0.84	**< 0.01**	0.76
PL	60.6 ± 7.4	60.4 ± 8.1	130.6 ± 12.6	140.4 ± 12.5	146.4 ± 13.9	150.9 ± 13.1	154.0 ± 13.6	155.3 ± 13.0			
RPE (N/A)				c	cd	cde	cde	cdef			
EAA			12.2 ± 0.8	13.2 ± 1.1	13.8 ± 1.2	14.3 ± 1.3	14.4 ± 1.2	14.6 ± 1.3	0.90	**< 0.01**	0.19
PL			12.2 ± 1.2	12.9 ± 1.0	13.7 ± 1.2	14.1 ± 1.5	14.6 ± 1.6	14.9 ± 1.9			
Glucose (mg/dl)											
EAA	89.6 ± 7.0	86.4 ± 5.3						87.0 ± 6.5	0.20	0.35	0.15
PL	88.8 ± 6.3	88.5 ± 5.6						89.3 ± 8.3			
Lactate (mM)		a						ab			
EAA	1.5 ± 0.3	1.7 ± 0.4						4.4 ± 2.0	0.93	**< 0.01**	0.88
PL	1.6 ± 0.3	1.7 ± 0.3						4.4 ± 2.5			
BDNF (pg/ml)		a						a			
EAA	23557.2 ± 7448.1	26241.4 ± 7875.5						28783.1 ± 8485.3	0.44	**< 0.01**	0.59
PL	22271.2 ± 8775.7	26559.4 ± 7529.3						27302.0 ± 6990.6			
1-MetHis (μmol/l)											
EAA	7.5 ± 7.3	7.7 ± 7.5						7.0 ± 7.0^ab^	0.60	**< 0.01**	**0.03**
PL	6.9 ± 5.0	6.8 ± 5.1						5.9 ± 4.4^ab^			
3-MetHis (μmol/l)											
EAA	4.4 ± 0.8	4.6 ± 0.8^a^						4.4 ± 0.9^b^	0.29	**< 0.01**	**0.02**
PL	4.4 ± 0.8	4.4 ± 0.8						4.2 ± 0.7^ab^			
Ala (μmol/l)		a						ab			
EAA	367.1 ± 86.9	395.7 ± 84.9						448.8 ± 78.0	0.92	**< 0.01**	0.22
PL	363.7 ± 62.6	387.6 ± 63.2						455.1 ± 67.5			
a-ABA (μmol/l)		a						ab			
EAA	19.3 ± 4.1	20.1 ± 3.9						17.3 ± 3.5	0.62	**< 0.01**	0.48
PL	19.7 ± 4.7	20.3 ± 4.7						17.9 ± 4.0			
Arg (μmol/l)											
EAA	97.7 ± 15.0	107.0 ± 17.8^a^						100.1 ± 12.2^b^	0.02	**< 0.01**	**< 0.01**
PL	96.3 ± 19.2	98.5 ± 19.2						91.3 ± 15.0^b^			
Asn (μmol/l)											
EAA	49.2 ± 6.0	52.6 ± 6.4^a^						43.1 ± 5.2^ab^	0.83	**< 0.01**	**< 0.01**
PL	48.9 ± 7.1	51.3 ± 7.2^a^						45.3 ± 6.2^ab^			
Asp (μmol/l)		a						b			
EAA	2.2 ± 0.7	2.0 ± 0.6						2.4 ± 0.5	0.70	**< 0.01**	0.19
PL	2.4 ± 1.0	1.8 ± 0.6						2.5 ± 0.7			
b-AiBA (μmol/l)											
EAA	1.7 ± 1.1	1.7 ± 1.1						1.8 ± 1.2	0.97	0.16	0.82
PL	1.8 ± 1.2	1.7 ± 1.1						1.8 ± 1.2			
Cit (μmol/l)											
EAA	29.5 ± 4.2	27.5 ± 4.0^a^						27.6 ± 4.4^a^	**0.02**	**< 0.01**	**< 0.01**
PL	28.6 ± 4.6	26.8 ± 4.3^a^						24.8 ± 4.0^ab^			
Cys2 (μmol/l)		a						a			
EAA	24.5 ± 3.1	23.9 ± 3.8						23.0 ± 3.8	0.75	**< 0.01**	0.29
PL	24.6 ± 3.8	23.2 ± 3.7						22.9 ± 3.1			
EtOHNH2 (μmol/l)								ab			
EAA	7.7 ± 1.1	7.9 ± 1.0						9.2 ± 1.3	0.26	**< 0.01**	0.58
PL	7.9 ± 1.2	8.0 ± 1.2						9.5 ± 1.3			
Glu (μmol/l)		a						ab			
EAA	29.1 ± 15.8	22.8 ± 10.7						39.3 ± 8.4	0.86	**< 0.01**	0.30
PL	28.7 ± 15.3	20.8 ± 8.3						41.0 ± 12.0			
Gln (μmol/l)		a						b			
EAA	599.9 ± 57.6	644.2 ± 66.4						598.1 ± 52.3	0.11	**< 0.01**	0.09
PL	597.5 ± 66.3	626.5 ± 75.0						581.2 ± 54.5			
Gly (μmol/l)											
EAA	252.2 ± 53.8	265.5 ± 60.2^a^						226.2 ± 50.1^ab^	0.30	**< 0.01**	**< 0.01**
PL	241.1 ± 46.1	253.6 ± 51.5^a^						231.5 ± 39.4^b^			
His (μmol/l)											
EAA	80.3 ± 7.3	84.4 ± 7.2^a^						78.9 ± 5.8^b^	0.14	**< 0.01**	**0.01**
PL	79.7 ± 6.0	80.8 ± 6.9						77.1 ± 6.0^b^			
HyPro (μmol/l)		a						ab			
EAA	18.6 ± 7.7	20.0 ± 7.9						16.3 ± 6.2	0.27	**< 0.01**	0.25
PL	16.8 ± 5.4	17.8 ± 5.6						14.9 ± 4.2			
Ile (μmol/l)											
EAA	73.6 ± 12.5	117.7 ± 29.8^a^						86.0 ± 15.0^ab^	**< 0.01**	**< 0.01**	**< 0.01**
PL	75.3 ± 11.0	75.7 ± 15.2						70.9 ± 10.2^a^			
Leu (μmol/l)											
EAA	139.0 ± 18.9	275.7 ± 81.4^a^						236.9 ± 40.3^a^	**< 0.01**	**< 0.01**	**< 0.01**
PL	137.8 ± 16.2	145.2 ± 45.7						134.5 ± 25.2			
Lys (μmol/l)											
EAA	194.0 ± 30.5	252.1 ± 46.5^a^						223.4 ± 25.5^ab^	**< 0.01**	**< 0.01**	**< 0.01**
PL	190.8 ± 33.1	200.3 ± 31.0						184.2 ± 27.0^b^			
Met (μmol/l)											
EAA	29.1 ± 2.9	38.2 ± 5.0^a^						31.2 ± 2.5^ab^	**< 0.01**	**< 0.01**	**< 0.01**
PL	28.2 ± 3.1	28.8 ± 3.8						27.1 ± 3.5^b^			
Orn (μmol/l)											
EAA	48.5 ± 8.7	50.1 ± 8.7^a^						46.5 ± 7.9^ab^	0.13	**< 0.01**	**< 0.01**
PL	48.4 ± 8.2	48.6 ± 7.9						43.6 ± 7.1^ab^			
Phe (μmol/l)											
EAA	59.4 ± 5.7	72.8 ± 8.8^a^						64.3 ± 5.3^ab^	**< 0.01**	**< 0.01**	**< 0.01**
PL	58.6 ± 5.5	61.6 ± 7.1^a^						59.5 ± 6.3^b^			
Pro (μmol/l)											
EAA	174.3 ± 61.7	182.1 ± 62.6^a^						167.2 ± 57.6^ab^	0.49	**< 0.01**	**0.05**
PL	167.1 ± 48.7	174.6 ± 49.6^a^						166.0 ± 47.8^b^			
Sar (μmol/l)											
EAA	2.3 ± 0.6	2.6 ± 0.7^a^						2.3 ± 0.6^b^	0.39	**< 0.01**	**0.01**
PL	2.3 ± 0.7	2.4 ± 0.8^a^						2.3 ± 0.7			
Ser (μmol/l)											
EAA	122.5 ± 14.4	128.6 ± 17.9^a^						110.0 ± 16.1^ab^	0.10	**< 0.01**	**< 0.01**
PL	119.2 ± 17.3	120.0 ± 18.8						109.6 ± 15.3^ab^			
Tau (μmol/l)								ab			
EAA	54.0 ± 9.2	52.8 ± 9.6						63.9 ± 14.5	0.25	**< 0.01**	0.78
PL	52.6 ± 10	49.9 ± 11.1						62.2 ± 15.1			
Thr (μmol/l)											
EAA	133.9 ± 18.9	154.4 ± 19.7^a^						137.4 ± 13.4^b^	**< 0.01**	**< 0.01**	**< 0.01**
PL	129.1 ± 25.4	134.6 ± 24.9^a^						120.5 ± 17.2^ab^			
Trp (μmol/l)											
EAA	65.9 ± 7	70.3 ± 6.9^a^						60.3 ± 6.1^ab^	0.41	**< 0.01**	**0.03**
PL	65.6 ± 6.5	67.4 ± 7.5						59.4 ± 6.6^ab^			
Tyr (μmol/l)											
EAA	63.0 ± 8.7	65.3 ± 8.9^a^						59.5 ± 8.1^ab^	0.06	**< 0.01**	**< 0.01**
PL	61.4 ± 7.4	60.5 ± 7.9						57.7 ± 8.1^ab^			
Val (μmol/l)											
EAA	247.9 ± 37	300.9 ± 51.8^a^						270.2 ± 35.9^ab^	**< 0.01**	**< 0.01**	**< 0.01**
PL	252.4 ± 31.3	250.1 ± 31.7						236.9 ± 27.8^ab^			
Kyn (μmol/l)											
EAA	1.7 ± 0.2	1.8 ± 0.2^a^						1.7 ± 0.2^b^	0.10	**< 0.01**	**0.02**
PL	1.7 ± 0.3	1.8 ± 0.3^a^						1.8 ± 0.3			

Values are presented as mean ± SD. The p-values shown in the table represent the results of two-way analysis of variance, and the letters a–g represent the results of the comparison between time points by paired t-test with Bonferroni correction.

^a^*P* < 0.05 vs. Baseline; ^b^*P* < 0.05 vs. pre-EX; ^c^*P* < 0.05 vs. 5 min; ^d^*P* < 0.05 vs. 10 min; ^e^*P* < 0.05 vs. 15 min; ^f^*P* < 0.05 vs. 20 min; ^g^*P* < 0.05 vs. 25 min.

Significant values are in bold.

### Changes in color-word Stroop task (CWST)-measured EF and memory recognition task (MRT)-measured memory recognition (MR)

There was a significant main effect of time on the reaction times (RT) in all CWST tasks and the response accuracy (RA) in the incongruent task, indicating that the RTs in all CWST tasks were shorter at immediately after the completion of the exercise session (post-EX) (*P*s < 0.01, baseline and pre-EX vs. post-EX; Table 2) and that RA in the incongruent task was improved at post-EX (both *P*s < 0.01, baseline and before exercise (pre-EX) vs. post-EX; Table 2). In contrast, no significant main effect of treatment or significant treatment × time interaction (see Table 2) were observed.

**Table 2:** Cognitive tasks and psychological conditions throughout EAA and PL treatments.

	Time points			*P* values		
	Baseline	Pre-EX	Post-EX	Treatment	Time	Interaction
Color-word Stroop tasks						
Reaction time (ms)						
Congruent task			ab			
EAA	9273 ± 1500	9364 ± 1553	8673 ± 1521	0.82	**< 0.01**	0.80
PL	9287 ± 1735	9225 ± 1653	8677 ± 1630			
Neutral task			ab			
EAA	9662 ± 1766	9852 ± 1722	9024 ± 1333	0.40	**< 0.01**	0.27
PL	10015 ± 2166	9929 ± 1952	9076 ± 1661			
Incongruent task			ab			
EAA	10703 ± 2251	10621 ± 1916	9441 ± 1332	0.26	**< 0.01**	0.53
PL	10807 ± 2370	10793 ± 2318	9791 ± 1848			
Response accuracy (%)						
Congruent task						
EAA	96 ± 4	97 ± 2	97 ± 3	0.20	0.19	0.80
PL	97 ± 3	97 ± 3	98 ± 2			
Neutral task						
EAA	97 ± 2	97 ± 3	97 ± 3	0.74	0.83	0.80
PL	98 ± 3	97 ± 3	97 ± 3			
Incongruent task			ab			
EAA	96 ± 4	96 ± 4	98 ± 2	0.56	**< 0.01**	0.23
PL	97 ± 3	96 ± 3	97 ± 3			
Reverse-Stroop interference score (%)			ab			
EAA	12.4 ± 6.5	9.8 ± 8.5	4.4 ± 4.6	0.94	**< 0.01**	0.06
PL	9.2 ± 6.6	9.5 ± 5.0	8.1 ± 7.1			
Memory recognition task						
Reaction time (ms)						
EAA	812 ± 103		809 ± 104	0.57	0.30	0.15
PL	802 ± 128		831 ± 92			
Response accuracy (%)			a			
EAA	81 ± 8		77 ± 7	0.55	**< 0.01**	0.38
PL	81 ± 7		79 ± 8			
Arousal (N/A)			ab			
EAA	2.7 ± 0.8	2.9 ± 0.7	4.0 ± 0.8	0.89	**< 0.01**	0.85
PL	2.7 ± 0.9	3.0 ± 0.9	3.9 ± 0.9			
Mental fatigue (mm)			ab			
EAA	23 ± 18	24 ± 20	35 ± 22	0.56	**< 0.01**	0.84
PL	25 ± 22	27 ± 21	35 ± 28			
Concentrate (mm)						
EAA	61 ± 20	63 ± 18	74 ± 20	0.30	**< 0.01**	0.52
PL	59 ± 23	62 ± 22	68 ± 24			
Motivation (mm)						
EAA	69 ± 21	71 ± 19	75 ± 18	0.69	0.09	0.58
PL	69 ± 22	70 ± 21	73 ± 20			

Values are presented as mean ± SD. The p-values shown in the table represent the results of two-way analysis of variance, and the letters a–g represent the results of the comparison between time points by paired t-test with Bonferroni correction.

^a^*P* < 0.05 vs. Baseline; ^b^*P* < 0.05 vs. pre-EX; ^c^*P* < 0.05 vs. 5 min; ^d^*P* < 0.05 vs. 10 min; ^e^*P* < 0.05 vs. 15 min; ^f^*P* < 0.05 vs. 20 min; ^g^*P* < 0.05 vs. 25 min.

Significant values are in bold.

**Figure 1:**
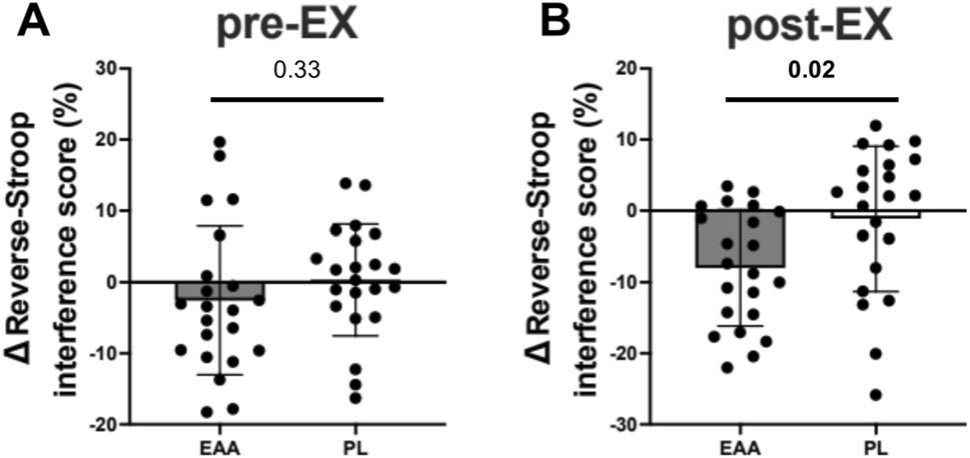
Comparison of changes between EAA and PL treatment in the reverse-Stroop interference scores. Comparison of changes from baseline to each time point (∆) between EAA and PL treatments in the reverse-Stroop interference scores. Gray indicates participants who received EAA supplements, and white indicates participants who received the PL. The values are means ± SD. (**A**) Means ± SD and individual data (black circles) of changes in the reverse-Stroop interference score from baseline to before exercise (i.e., post-EX-aseline). (**B**) Means ± SD and individual data (black circles) of changes in the reverse-Stroop interference score from baseline to after exercise (i.e., post-EX-baseline).  The p-values shown above the bar represent the results of comparisons between treatments by paired t-test. Significant values are in bold.

There was a significant main effect of time (*P* < 0.01) on the reverse-Stroop interference score; scores improved at post-EX after both EAA and placebo (PL) treatments (*P*s < 0.01, baseline and pre-EX vs. post-EX). No significant main effect of treatment (*P* = 0.94) were observed. Regarding the interaction between treatment × time, the result of the repeated-measures analysis of variance (ANOVA) test was not significant (*P* = 0.06) but was suggestive that there may be a certain degree of association (see Table 2). Therefore, given the low power of the test for interaction in the ANOVA, we conducted an ad-hoc analysis for the amount of change from baseline on the reverse-Stroop interference score. This analysis indicated that the improvement on the reverse-Stroop interference score at post-EX after EAA treatment was greater than that after PL treatment (*P* = 0.02; Fig. 1), although there was no difference at pre-EX (*P* = 0.33).

For the RA on the MRT, there was a significant main effect of time (*P* < 0.01, baseline vs. post-EX; Table 2), indicating that the RA decreased after exercise after both treatments. However, no significant main effect of treatment (*P* = 0.55) or significant treatment × time interaction (*P* = 0.38) were observed. Analyses of RT also revealed no significant main effects of time (*P* = 0.30) or treatment (*P* = 0.57) and no significant interaction (*P* = 0.15) effect.

### Changes in the rating of perceived exertion (RPE), heart rate (HR), and blood metabolites

There were significant main effects of time on RPE (*P* < 0.01) and HR (*P* < 0.01), although the elevations in RPE (*P* = 0.90) and HR (*P* = 0.84) during exercise were not significantly different between the two treatments.

There was a significant main effect of time on blood lactate (*P* < 0.01), indicating that blood lactate increased after exercise after both the EAA and PL treatments (both *P*s < 0.01, baseline and pre-EX vs. post-EX; Table 1). However, no significant main effect of treatment (*P* = 0.93) or significant treatment × time interaction (*P* = 0.88) were observed.

Analyses of blood glucose also revealed no significant main effects of time (*P* = 0.35) or treatment (*P* = 0.20) and no significant interaction (*P* = 0.15) effect.

### Changes in plasma amino acid levels

**Figure 2:**
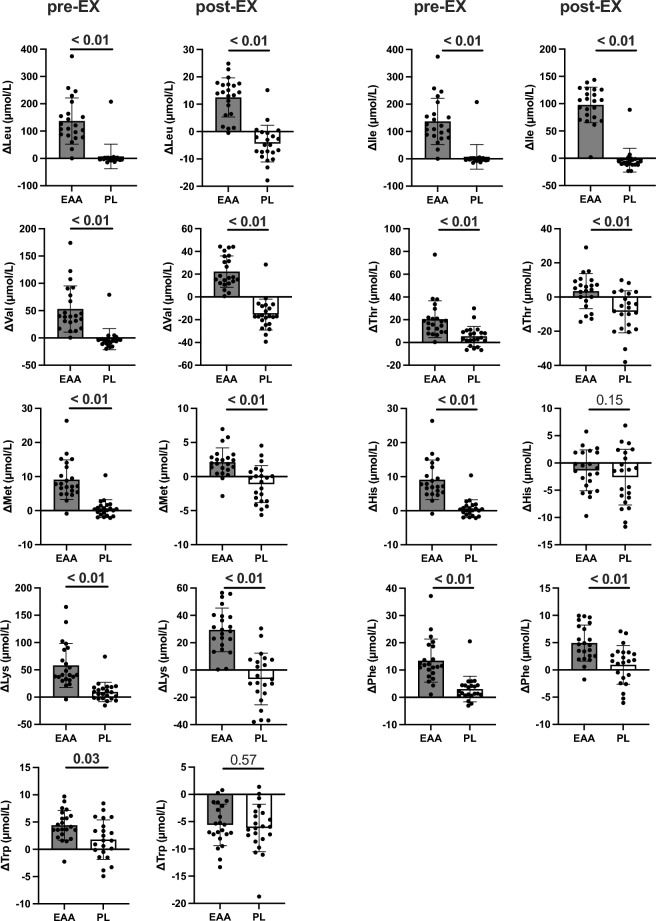
Changes in amino acid levels in participants who received the EAA supplements. The extent of changes from baseline to each time point in amino acid levels in participants who received the EAA supplements (gray) and those who received the PL (white). The values are means ± SD. The p-values shown above the bar represent the results of comparisons between treatments by paired t-test. Significant values are in bold.

There was a significant main effect of time on the plasma levels of all amino acids that participants received (i.e., leucine (Leu), isoleucine (Ile), valine (Val), threonine (Thr), methionine (Met), histidine (His), lysine (Lys), phenylalanine (Phe), and tryptophan (Trp)) (*P*s < 0.01; Table 1). Analyses of the plasma levels of Leu, Ile, Val, Thr, Met, Lys, and Phe revealed a significant main effect of treatment (*P*s < 0.01) and a significant interaction effect (*P*s < 0.05). Analyses of the plasma levels of His and Trp also revealed a significant interaction effect (*P* = 0.01 for His; *P* = 0.03 for Trp) but no significant main effect of treatment (*P* = 0.14 for His; *P* = 0.41 for Trp). Plasma levels of all amino acids that participants received increased 15 min after ingestion of EAA supplements (*P*s < 0.01, baseline vs. pre-EX). The plasma levels of Phe and Thr at pre-EX were also higher than those at baseline after PL treatment (*P* = 0.02 for Phe;* P* = 0.03 for Thr). The extent of changes from baseline to pre-EX in the level of each essential amino acid was higher after EAA treatment than that after PL treatment (*P*s < 0.05; Fig. 2). The plasma level of Leu at post-EX did not differ from that before exercise after either the EAA or PL treatments (*P*s < 0.05, baseline and/or pre-EX vs. post-EX). The extent of changes from baseline to post-EX in the level of each essential amino acid (excluding His and Trp) was higher after EAA treatment than that after PL treatment (*P*s < 0.01).

There was a significant main effect of time (*P* < 0.01; Table 1) on the plasma levels of glutamine (Gln), but no significant main effect of treatment (*P* = 0.11) and a significant interaction effect (*P* = 0.09). For both treatments, plasma levels of Gln increased immediately before exercise (both *P*s < 0.01, baseline vs. pre-EX) and reduced after exercise compared to immediately before exercise (both *P*s < 0.01, pre-EX vs. post-EX). There was a significant main effect of time and treatment (both *P*s < 0.05) and a significant interaction effect (*P* < 0.01) on the plasma levels of arginine (Arg). Plasma levels of Arg increased 15 min after ingestion of EAA supplements (*P* < 0.01, baseline vs. pre-EX) and reduced after exercise compared to immediately before exercise in both conditions (both *P*s < 0.01, pre-EX vs. post-EX). The extent of changes from baseline to pre-EX and post-EX in the level of Arg was higher after EAA treatment than that after PL treatment (*P*s < 0.05; Table 3). There was a significant main effect of time (*P* < 0.01) and a significant interaction effect (*P* = 0.02) on the plasma levels of 3-MetHis, but no significant main effect of treatment (*P* = 0.29). Plasma levels of 3-MetHis increased 15 min after ingestion of EAA supplements (*P* < 0.01, baseline vs. pre-EX) and reduced after exercise compared to before exercise in both conditions (both *P*s < 0.05, baseline and/or pre-EX vs. post-EX). The extent of changes from baseline to pre-EX in the level of 3-MetHis was higher after EAA treatment than that after PL treatment (*P* = 0.02). There was a significant main effect of time (*P* < 0.01) and a significant interaction effect (*P* = 0.02) on the plasma levels of kynurenine (Kyn), but no significant main effect of treatment (*P* = 0.10). Plasma levels of Kyn increased immediately before exercise for both treatments (both *P*s < 0.05, baseline vs. pre-EX) and reduced after exercise compared to immediately before exercise in EAA treatments (*P* = 0.02, pre-EX vs. post-EX). The extent of changes from baseline to post-EX in the level of Kyn was lower after EAA treatment than that after PL treatment (*P* = 0.04).

**Table 3:** The contrast between EAA versus PL deltas for blood data.

	Treatments		*P* value
	EAA	PL	
Δ1-MetHis (μmol/l)			
Pre-EX	−0.19 ± 0.38	−0.14 ± 0.28	**< 0.01**
30 min (i.e., post-EX)	−0.51 ± 0.56	−0.95 ± 0.68	**0.02**
Δ3-MetHis (μmol/l)			
Pre-EX	−0.18 ± 0.27	−0.01 ± 0.26	**0.02**
30 min (i.e., post-EX)	−0.03 ± 0.35	−0.20 ± 0.30	0.10
ΔArg (μmol/l)			
Pre-EX	−9.33 ± 9.91	−2.22 ± 4.65	**< 0.01**
30 min (i.e., post-EX)	−2.41 ± 8.52	−4.99 ± 10.49	**0.01**
ΔAsn (μmol/l)			
Pre-EX	−3.38 ± 2.44	−2.39 ± 2.58	0.20
30 min (i.e., post-EX)	−6.18 ± 3.86	−3.61 ± 4.2	**0.04**
ΔCit (μmol/l)			
Pre-EX	−2.04 ± 1.07	−1.74 ± 1.25	0.40
30 min (i.e., post-EX)	−1.87 ± 2.60	−3.80 ± 2.52	**0.02**
ΔGly (μmol/l)			
Pre-EX	−13.33 ± 15.54	−12.41 ± 15.08	0.84
30 min (i.e., post-EX)	−25.99 ± 14.63	−9.62 ± 20.17	**0.01**
ΔOrn (μmol/l)			
Pre-EX	−1.59 ± 2.37	−0.19 ± 1.80	**0.03**
30 min (i.e., post-EX)	−2.01 ± 2.46	−4.81 ± 2.65	**< 0.01**
ΔPro (μmol/l)			
Pre-EX	−7.84 ± 8.54	−7.49 ± 8.81	0.90
30 min (i.e., post-EX)	−7.11 ± 12.31	−1.08 ± 12.34	0.11
ΔSar (μmol/l)			
Pre-EX	−0.30 ± 0.22	−0.13 ± 0.19	**0.01**
30 min (i.e., post-EX)	−0.04 ± 0.16	−0.04 ± 0.17	0.94
ΔSer (μmol/l)			
Pre-EX	−6.07 ± 6.91	−0.79 ± 5.49	**0.01**
30 min (i.e., post-EX)	−12.48 ± 7.51	−9.62 ± 7.54	0.21
ΔTyr (μmol/l)			
Pre-EX	−2.31 ± 4.37	−0.89 ± 2.71	**0.01**
30 min (i.e., post-EX)	−3.58 ± 3.66	−3.72 ± 3.81	0.90
ΔKyn (μmol/l)			
Pre-EX	−0.07 ± 0.12	−0.09 ± 0.10	0.65
30 min (i.e., post-EX)	−0.02 ± 0.13	−0.06 ± 0.14	**0.04**

Values are presented as mean ± SD. The p-values shown in the table represent the results of comparisons between treatments by paired t-test.

Significant values are in bold.

Changes in the levels of amino acids not included in EAA supplements and amino acid metabolites are shown in Tables 1 and 3.

### Changes in psychological conditions for cognitive tasks

There was a significant main effect of time on arousal (*P* < 0.01; Table 2) but not a significant main effect of treatment (*P* = 0.89) or a significant treatment × time interaction (*P* = 0.85). Arousal was higher at post-EX than at baseline (*P* < 0.01) and pre-EX (*P* < 0.01). There was a significant main effect of time on mental fatigue (*P* < 0.01) but not a significant main effect of treatment (*P* = 0.56) or a significant treatment × time interaction (*P* = 0.84). Mental fatigue was higher at post-EX compared to baseline (*P* < 0.05) and pre-EX (*P* < 0.05). However, while there was a significant main effect of time on the ability to concentrate (*P* < 0.01), post hoc comparisons showed no significant effect of exercise. There were no changes in motivation throughout either treatment.

### Relationships between the plasma levels of each amino acid and the reverse-Stroop interference score

**Figure 3:**
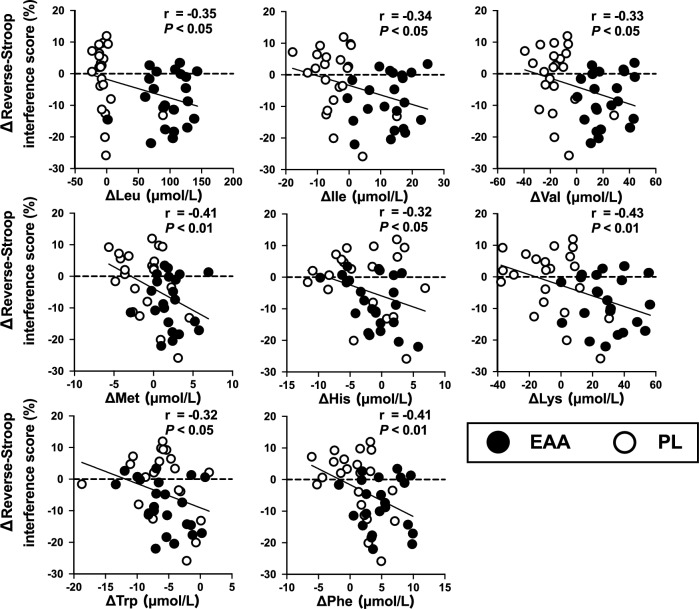
Relationship between the extent of change from baseline to post-EX for the reverse-Stroop interference scores and the level of each amino acid after EAA treatment.

Figure 3 shows the relationship between the changes in levels of each essential amino acid and the changes in EF from baseline to post-EX. The changes in the reverse-Stroop interference score were correlated with changes in the levels of Leu (r = -0.35, *P* < 0.05), Ile (r = -0.34, *P* < 0.05), Val (r = -0.33, *P* < 0.05), Met (r = -0.41, *P* < 0.01), His (r = -0.32, *P* < 0.05), Lys (r = -0.43, *P* < 0.01), Trp (r = -0.32, < 0.05), and Phe (r = -0.41, *P* < 0.01). Correlations were also observed between the reverse-Stroop interference score and Gln (r = -0.34, *P* < 0.05), Arg (r = -0.46, *P* < 0.01) and 3-MetHis (r = -0.37, *P* < 0.05), which were not included in the EAA supplements.

## Discussion

Consistent with previous findings^7^, the present study demonstrated that 30 min of moderate-intensity aerobic exercise improved EF. Moreover, ad-hoc analysis showed that EF after EAA treatment and exercise was greater than that following PL treatment and exercise. In addition, the improvement in EF was correlated with increases in plasma levels of amino acids (leucine, isoleucine, valine, lysine, and phenylalanine), which are potential substrates for synthesizing neurotransmitters in the brain. In contrast, EAA ingestion did not affect MR or serum BDNF levels throughout the experiment. MR was impaired immediately after moderate-intensity aerobic exercise. Serum BDNF was increased before exercise, and the increase in BDNF was continued throughout the experiment. Taken together, these results suggest that EAA supplements ingestion before exercise had a positive effect on EF.

Although much is known regarding the positive effects of nutrition and acute exercise on muscle protein synthesis^19^, little is known about the effects of nutrition and acute exercise on cognitive function. The present findings showed that BCAA supplements ingestion before moderate-intensity aerobic exercise was effective in improving EF. Similar to the present study, the ingestion of BCAA was previously shown to shorten the choice reaction time during aerobic exercise^12^. However, the previous study did not measure blood levels of amino acids; thus, the underlying mechanisms remained unclear. In contrast, we are the first to demonstrate that an increase in levels of amino acids, the precursors of neurotransmitters, may be a potential mechanism by which the ingestion of an EAA supplements improves EF. EF is modulated by neurotransmitters (i.e., dopamine, norepinephrine, and glutamate)^20–22^. Indeed, an increase in neurotransmitter levels acutely improves cognitive function, including EF^23,24^. Plasma levels of Leu, Ile, Val, Lys, Phe and Met 15 min after supplement ingestion and after exercise were higher for EAA treatment than for PL treatment. Moreover, higher plasma levels of these amino acids were associated with better EF after exercise. These amino acids (Leu, Ile, Val, Lys, Phe, and Met) are precursors for neurotransmitters (dopamine, norepinephrine, and glutamate)^25–29^. Brain glutamate is synthesized from Leu and Lys^25,26^. Val and Ile are also involved in the synthesis of glutamate^26^. Dopamine and norepinephrine are synthesized from Tyr, whose precursor is Phe^27^. The intake of *S*-adenosyl-L-methionine, which is synthesized in the body from Met, increases dopamine and norepinephrine in the brain^29^. Then, the rate of synthesis and release of these neurotransmitters is thought to be directly modulated by brain concentrations of their amino acid precursors, BCAA, Phe and Lys, and Met is also influenced by their availability from the blood^27–29^. To our best of knowledge, there are no studies that investigate whether supplementation of Leu, Ile, Val, Lys, Phe and Met acutely increases neurotransmitters (dopamine, norepinephrine, and glutamate) in the brain. On the other hand, it has been shown that acute ingestion of glutamine, an amino acid precursor for brain neurotransmitter γ-aminobutyric acid (GABA) synthesis, acutely increases the brain neurotransmitter^30^. Thus, changes in peripheral amino acid concentrations due to amino acid supplementation can rapidly alter the dynamics of neurotransmitters in the brain. Collectively, we suggest that the intake of EAA supplement potentially augments neurotransmitters in the brain as precursors of them, and might have a positive effect on cognition after aerobic exercise. Mechanisms underlying acute exercise- and/or nutrition-induced improvements in cognition have been comprehensively studied^5,31,32^, and changes in neurotransmitters are one of the factors^31^. However, because of technical challenges, few studies have examined acute neurotransmitter changes in the human brain and hence their direct effect on cognitive function^31^. Nonetheless, our results provide important support of the idea that the neurotransmitter changes induced by EAA may positively affect cognition after aerobic exercise and highlight the importance of pairing EAA supplementation with exercise.

Regarding other amino acids, the plasma concentration of Arg was higher immediately after exercise in the EAA treatment compared to the PL treatment. Moreover, a significant negative correlation was also found between EF and the change in plasma Arg concentration. In other words, higher plasma Arg concentrations were associated with better EF at post-EX. Since Arg is the main precursor of nitric oxide (NO)^33^, it is inferred that NO synthesis via Arg increased under EAA treatment. Transient enhancement of NO synthesis enhances cognitive function^34^, so the enhancement of EF by intake of EAA supplement may involve enhancement of Arg-mediated NO synthesis.

No difference between treatments was observed in the plasma levels of Trp, His and Gln at post-EX. However, plasma levels of these amino acids were associated with better EF after exercise. Trp, His and Gln are precursors of the neurotransmitters (serotonin, histamine and GABA)^35–37^. In fact, by lowering available Trp in the plasma, serotonin in the brain decreases, leading to decreased EF^38^. GABA is crucial for effective information processing in the brain^39^, and ingestion of Gln, an amino acid precursor for brain neurotransmitter GABA synthesis, increases the brain neurotransmitter^30^. Therefore, regardless of the presence or absence of EAA supplementation, plasma level of Trp, His and Gln may be involved in improving EF after exercise through changes in neurotransmitters.

The relationship between amino acids metabolites and EF was evaluated because further insights could be gained by examining the relationship between these metabolites and EF. 3-methyl-histidine are metabolites of the His in the EAA supplements employed in this study. The 3-methyl-histidine was significantly correlated with improved EF after exercise. However, it is difficult to explain why 3-methyl-histidine was associated with the improvement in EF after exercise. Further research is needed to explain this event. Kyn can easily cross the blood–brain barrier, and it is thought to cause a decline in cognitive function through the inflammatory effects of Kyn metabolites in the brain^40^. Meanwhile, exercise increases circulation Kyn and may change the Kyn metabolites in the central nervous system^41^. However, it is unclear whether alternation of Kyn metabolism caused by exercise affects EF, and the interaction with EAA supplement ingestion is also unknown. Then, the relationship between changes in Kyn and changes in EF after exercise or EAA supplement ingestion was assessed. In EAA treatment, Kyn decreased after exercise compared to immediately before exercise (pre-EX vs post-EX). Moreover, the extent of changes from baseline to post-EX in the level of Kyn was lower after EAA treatment than that after PL treatment. Given an anti-inflammatory effect of EAA^42^, it may be reasonable to speculate that EAA treatment-reduced Kyn-mediated inflammation was favorable for the positive effect of EAA supplements ingestion before exercise on improved EF. However, it is difficult to conclude a direct relationship from this study, and further investigation is required.

The present study showed that moderate-intensity aerobic exercise had a negative impact on the RA of memory recognition. A previous meta-analysis showed that aerobic exercise improves short-term memory, and, importantly, that the positive effects of acute exercise on short-term memory were greater when aerobic exercise was performed for a short duration (< 20 min)^43^. In addition, Hacker and colleagues reported better RA after 15 min of aerobic exercise compared to after 30 min or 45 min^44^. Therefore, we surmise that memory recognition was impaired because the subjects performed 30 min of moderate-intensity aerobic exercise in this study.

Acute administration of BCAAs transiently increased BDNF levels in the rat brain^18^. In the present study, the ingestion of an EAA supplements mainly composed of BCAA did not affect serum BDNF levels in humans. Moreover, EAA supplements ingestion did not further enhance the increases in serum BDNF levels induced by moderate-intensity aerobic exercise. Our results showed that, unlike brain BDNF levels in rats, there were no changes in serum BDNF levels in humans in response to EAA ingestion.

Serum BDNF was increased at the time of pre-exercise, and the increase in BDNF was continued throughout the experiment. Given that psychological stress acutely increases BDNF^45,46^, and Stroop task conducted in this study is used as a method to induce psychological stress at the laboratory^47^, it is plausible that BDNF increased due to the Stroop task-induced psychological stress. Serum BDNF levels were not significantly different before and after exercise, indicating that these levels did not change in response to moderate-intensity aerobic exercise. A previous study demonstrated that the magnitude of the increase in serum BDNF levels in response to aerobic exercise is dependent on exercise intensity^48^. Consistent with the findings of the present study, serum BDNF levels were not increased by acute moderate-intensity aerobic exercise in Japanese men^49^. Circulating BDNF exists in two distinct pools: BDNF bound to platelets and BDNF circulating freely in plasma^50,51^. Blood serum measures represent the total measurable circulating BDNF, while plasma measures represent only the free portion^50,51^. In addition, a meta-analysis indicated that acute exercise enhanced BDNF in plasma to a greater extent than that in serum^50^. To fully characterize the BDNF response to acute exercise and EAA supplements ingestion, future studies should collect serum, plasma, and platelets and calculate the amount of BDNF per platelet^51^.

### Perspective

It is important to consider strategies to improve EF, which supports skills essential for mental and physical health^52^. However, the present study is the first to report that EAA ingestion before aerobic exercise may improve EF. Further studies are needed to investigate the chronic effect of a combination of EAA ingestion and aerobic exercise on EF to improve quality of life.

Chronic administration of EAAs may have clinical/physiological relevance in terms of their impact on both brain and skeletal muscle. Although the exact mechanism underlying skeletal muscle weakness and cognitive function remains unclear, age-related skeletal muscle weakness, including sarcopenia, is associated with decreased cognitive function^53^. EAA supplementation facilitates acute robust stimulation of muscle protein synthesis in older women^54^. Moreover, chronic intervention consisting of EAA supplementation and low-intensity resistance training increased muscle mass and strength in patients with sarcopenia^55^. Therefore, chronic EAA supplementation and exercise may be an effective strategy to treat not only skeletal muscle weakness but also EF decline in elderly individuals, including those with skeletal muscle weakness. Thus, examining the effect of EAA supplementation and aerobic exercise on EF in older individuals and patients with chronic disease (e.g., sarcopenia) is warranted based on our findings in healthy young men to corroborate the effectiveness of EAA supplementation and aerobic exercise in the clinical setting.

### Limitations

In this study, we found that EAA supplements ingestion has a positive effect on EF after moderate-intensity aerobic exercise. However, it should be noted that this study has some limitations. First, we did not set an intervention group that took EAA supplements and did not take exercise. Evaluating the effects of EAA ingestion alone would advance our understanding of the combined effect of exercise and EAA ingestion. Second, some of our conclusions are supported by ad-hoc statistical analyses, even though they should ideally be validated by a pre-designed analysis given the statistical multiplicity and the type-1 error inflation. However, the results of our pre-planned analysis, ANOVA, are also considered to be adequately supportive of the conclusion, given that the statistical power of the interaction term is not high.

## Conclusion

Our results suggested that EAA supplements ingestion has a positive effect on EF after moderate-intensity aerobic exercise, while MR and serum BDNF levels were not affected. An increase in amino acids, the precursors of neurotransmitters, may be a potential mechanism by which the ingestion of an EAA supplements improves EF. Therefore, we propose that the ingestion of EAAs before aerobic exercise may be a strategy to improve EF.

## Methods

### Participants

Twenty-two healthy young men (age: 22 ± 2 years, body height: 175 ± 5 cm, body weight: 66 ± 8 kg, peak oxygen uptake [*V*O_2_ peak]: 42 ± 5 ml/min/kg) were informed of the experimental procedures and potential risks and provided written informed consent to participate in the study. No participants had any known neurologic, cardiovascular, or pulmonary disorders; color blindness; or abnormal vision. The participants were instructed to avoid strenuous physical activity and abstain from caffeine and alcohol for 24 h before each experimental treatment. Moreover, the participants also abstained from food for 12 h (overnight fasting) before each experiment and were not taking any medications that would affect cognitive function. This study was conducted according to the guidelines of the Declaration of Helsinki. All procedures were approved by the Ethics Committee of Ritsumeikan University (BKC-IRB-2020-018) and Ajinomoto Co., Inc. (2020-004). The study was registered in the University Hospital Medical Information Network Clinical Trials Registry as UMIN000042638.

### Experimental procedure

Before the day of the experiment, all participants practiced both the CWST and MRT until they achieved consistent scores. Subsequently, the participants performed a ramp incremental test on a cycle ergometer to determine their *V*O_2_ peak. Afterward, the participants underwent experiments for 2 days (i.e., treatments) with a wash-out period of at least 1 week^12^.

**Figure 4:**
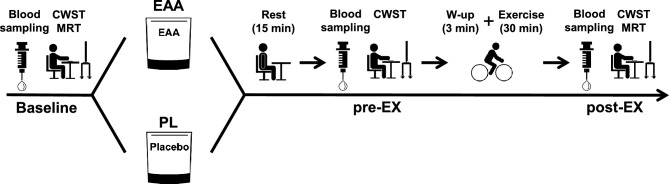
Overview of the experimental protocol.

On the experimental days, upon participant arrival, an 18-gauge cannula was placed in the cephalic vein of the arm for blood sampling. Subsequently, the participants also practiced the CWST again to minimize the learning effect. After resting in a seated upright position for at least 10 min, the baseline data were collected. Afterward, the participants received either an EAA supplements or the PL in a double-blind counterbalanced manner. Details on the supplements containing energy compounds with EAA and PL (Ajinomoto Co., Inc., Tokyo, Japan) are presented in Table 4. The collections of the data before exercise were started 15 min after supplements ingestion, and 30 min of moderate-intensity (i.e., 60% of the *V*O_2_ peak) cycling exercise with 3 min of warm-up at 100 W was started 30 min after supplements ingestion. During exercise, the participants were instructed to maintain a cadence of 60 rpm, which was carefully monitored by an examiner. Immediately after the completion of the exercise session, the data were collected (Fig. 4).

**Table 4:** Supplements composition of the essential amino acids (EAA) and the placebo (PL).

	EAA	PL
	g/4.7 g	g/4.7 g
l-Leucine	1.61	0.0
l-Isoleucine	0.43	0.0
l-Valine	0.44	0.0
l-Threonine	0.37	0.0
l-Methionine	0.13	0.0
l-Histidine	0.07	0.0
l-Lysine	0.67	0.0
l-Phenylalanine	0.27	0.0
l-Tryptophan	0.03	0.0
Maltitol	0.1	4.3
Activator, fragrance, etc	0.6	0.4

### Measurements

#### Peak oxygen uptake

A ramp incremental test was performed on a cycle ergometer to determine the *V*O_2_ peak of participants, which was used to calculate the workload for 60% of the *V*O_2_ peak. As previously described^56^, all participants performed 3 min of baseline cycling at 30 W, after which the workload was increased at a rate of 30 W/min until the limit of tolerance. The participants were asked to maintain a cadence of 60 rpm. During this test, breath-by-breath pulmonary gas-exchange data were collected and averaged every 30 s (AE-310S; Minato Medical Science, Osaka, Japan). The *V*O_2_ peak was determined as the highest 30-s mean value attained prior to exhaustion.

#### Cognitive functions

The CWST and MRT were administered to evaluate EF and memory recognition (MR), respectively. EF was measured before supplements ingestion, before exercise, and after exercise. MR was measured at baseline and post-EX.

##### Executive function

The CWST was administered to determine EF, as previously described^7,56^. In brief, for each task, 24 stimulus words that consisted of four color names (red, blue, green, and yellow, in Japanese characters) were randomly presented on a display. All participants repeatedly performed each of the three types of the CWST three times (i.e., nine times per test); the CWST types included congruent, neutral, and incongruent tasks (e.g., the word red presented in red, black, and blue/yellow/green font, respectively). The participants were instructed to press the color-labeled key that corresponded to the text meaning. The total RT of all 24 stimulus words and RA were collected for analysis. EF was assessed using the reverse-Stroop interference score, defined as the difference between the averaged RTs on the neutral and incongruent tasks^7,56^. The reverse-Stroop interference score was calculated as [(RT on the incongruent task − RT on the neutral task)/RT on the neutral task × 100]^57^.

##### Recognition memory

The evaluation of MR in this study was performed using the MRT^58^. First, in the memorization phase, all participants memorized 30 words from the Japanese language displayed for 1 s each. Afterward, the participants completed the CWST and waited to begin the MR phase (total: 5 min). To assess MR, 60 words (i.e., 30 of the memorized words and 30 distracters) were presented every 2 s in the MR phase. Participants pressed a button as quickly as possible to indicate words they believed they had memorized in the memorization phase; otherwise, they waited for the next question without doing anything. RA and RT were collected to evaluate MR. We calculated the RA as follows: RA (%) = Number of correct trials/60×100^58^. The recorded RTs on the correct trials were averaged. In other words, the recorded RTs on incorrect trials were excluded from analysis.

#### Blood metabolites

Blood samples were taken at baseline, pre-EX, and post-EX. Blood was collected in anticoagulant-free tubes (Terumo, Tokyo, Japan) for serum samples, tubes containing ethylene diamine tetraacetic acid-2Na (Terumo, Tokyo, Japan) for plasma samples, and 1-ml syringes for determination of blood glucose (Medisafe FIT Blood Glucose Meter; Terumo, Tokyo, Japan) and lactate concentrations (Lactate Pro 2; Arkray, Kyoto, Japan). The anticoagulant-free tubes were centrifuged (10 min at 3000 rpm, 4 °C) to obtain serum samples after 30 min at room temperature to allow clotting. The tubes containing EDTA-2Na were centrifuged (10 min at 3000 rpm, 4 °C) to obtain plasma after 5 min on ice. These samples were aliquoted and stored at −80 °C until analysis. Serum samples were used for the assessment of BDNF. The BDNF concentrations were determined with ELISA kits (DBNT00; R&D Systems, Minneapolis MN, USA). The intra- and interassay coefficients of variation for BDNF were < 3 and < 5%, respectively. Plasma samples were used for the assessment of each amino acid. The plasma amino acid concentrations were measured by high-performance liquid chromatography–electrospray ionization mass spectrometry (LC–MS) followed by precolumn derivatization as previously described^59^. Kynurenine (Kyn) was measured using LC–MS according to a previously reported method^60^. The following levels of 31 amino acids and kynurenine were quantified: 1-methyl-histidine (1-MetHis), 2-aminoethanol (EtOHNH_2_), 3-methyl-histidine (3-MetHis), alanine (Ala), alpha-aminobutyric acid (α-ABA), arginine (Arg), asparagine (Asn), aspartic acid (Asp), beta-aminoisobutyric acid (β-AiBA), citrulline (Cit), cysteine (Cys), gamma-aminobutyric acid (GABA), glutamate (Glu), glutamine (Gln), glycine (Gly), histidine (His), hydroxyproline (HyPro), isoleucine (Ile), leucine (Leu), lysine (Lys), methionine (Met), ornithine (Orn), phenylalanine (Phe), proline (Pro), sarcosine (Sar), serine (Ser), taurine (Tau), threonine (Thr), tryptophan (Trp), tyrosine (Tyr), and valine (Val).

#### Heart rate

To check the exercise intensity levels, HR was measured every 5 min (i.e., six times) during exercise via telemetry (RS400; Polar Electro Japan, Tokyo, Japan).

#### Psychological conditions

RPE was evaluated as the subjective intensity every 5 min during exercise, rated on Borg’s 15-point scale, which ranges from 6 (no exertion) to 20 (maximal exertion)^61^.

To assess the influence of arousal on cognitive function, the arousal level was measured immediately after the completion of the MRT using the Felt Arousal Scale (FAS), which is a 6-point, single-item scale ranging from 1 (low arousal) to 6 (high arousal)^62^. Similarly, a visual analog scale (VAS) was used to assess each of the following psychological conditions: mental fatigue, ability to concentrate, and motivation. Each VAS was labeled from 0 mm (not at all) to 100 mm (extremely), and participants drew lines on the VAS to indicate their psychological state during the cognitive tests^7,56^.

### Sample size

We have previously conducted a study similar to this protocol, which confirmed the effect of exercise and nutrient on cognitive function^7^. Based on the results of that study, which have reported the Cohen’s d effect size of the intervention on the CWST interference score to be 0.83, we conservatively estimated the expected effect size to be 0.7 in this study. Assuming the significance level of 5%, the power of at least 80%, and the intra-individual variability/inter-individual variability ratio = 1, the minimum number of subjects required for a 2 × 2 crossover study was calculated to be 18 subjects. Finally, 22 subjects were included after accounting for dropouts.

### Statistical analysis

All data are presented as the mean ± standard deviation. The time-series data were analyzed using two-way (treatment × time) repeated-measures analysis of variance (ANOVA). If the sphericity assumption was not met, Greenhouse–Geisser corrections were used. In addition, specific differences between timepoints were identified by paired *t* test with Bonferroni correction. To further examine the effects of EAA supplements on the CWST-measured EF and plasma amino acid and amino acid metabolites levels, for which possible effects of the intervention were suggested by ANOVA, the amount of change from baseline to pre- and post-EX each timepoint was tested between the EAA and the PL treatments with the paired t test. Since this was conducted as an ad-hoc analysis to provide exploratory insights on the possible effects of the EEA supplementation, no correction for multiplicity was applied. The statistical significance level was set at *P* < 0.05. Moreover, Pearson correlation analysis was used to analyze the relationship between the extent of change from baseline to post-EX in the reverse-Stroop interference score and each amino acid level. All statistical analyses were conducted using IBM SPSS software (Ver. 27.0, IBM Corp, NY, USA) and R software (ver. 4.0.2)^63^.

## Data Availability

The data that support the findings of this study are subject to general data protection regulations. Hence, not publicly available, but are available upon reasonable request from the corresponding author upon approval of the principal investigator.
